# Immunotherapy and Pancreatic Cancer: A Lost Challenge?

**DOI:** 10.3390/life13071482

**Published:** 2023-06-30

**Authors:** Carmelo Laface, Riccardo Memeo, Felicia Maria Maselli, Anna Natalizia Santoro, Maria Laura Iaia, Francesca Ambrogio, Marigia Laterza, Gerardo Cazzato, Chiara Guarini, Pierluigi De Santis, Martina Perrone, Palma Fedele

**Affiliations:** 1 Medical Oncology, Dario Camberlingo Hospital, 72021 Francavilla Fontana, Italy; 2 Unit of Hepato-Pancreatic-Biliary Surgery, “F. Miulli” General Regional Hospital, 70021 Acquaviva Delle Fonti, Italy; 3 Section of Dermatology, Department of Biomedical Science and Human Oncology, University of Bari, 70124 Bari, Italy; 4 Division of Cardiac Surgery, University of Bari, 70124 Bari, Italy; 5 Department of Emergency and Organ Transplantation, Pathology Section, University of Bari “Aldo Moro”, Piazza Giulio Cesare 11, 70124 Bari, Italy

**Keywords:** immunotherapy, pancreatic cancer, tumor microenvironment, TMB, immune biomarkers, PD-L1, PD-1, microsatellite instability, mismatch repair deficiency

## Abstract

Although immunotherapy has proved to be a very efficient therapeutic strategy for many types of tumors, the results for pancreatic cancer (PC) have been very poor. Indeed, chemotherapy remains the standard treatment for this tumor in the advanced stage. Clinical data showed that only a small portion of PC patients with high microsatellite instability/mismatch repair deficiency benefit from immunotherapy. However, the low prevalence of these alterations was not sufficient to lead to a practice change in the treatment strategy of this tumor. The main reasons for the poor efficacy of immunotherapy probably lie in the peculiar features of the pancreatic tumor microenvironment in comparison with other malignancies. In addition, the biomarkers usually evaluated to define immunotherapy efficacy in other cancers appear to be useless in PC. This review aims to describe the main features of the pancreatic tumor microenvironment from an immunological point of view and to summarize the current data on immunotherapy efficacy and immune biomarkers in PC.

## 1. Introduction

Although pancreatic cancer (PC) has a lower incidence with respect to other tumors, it corresponds to one of the leading causes of cancer death in the world [1]. In fact, it is the third highest cause of cancer-related death with a constantly increasing number of cases. The poor prognosis is mainly due to the late diagnosis, at an advanced stage, with only 3% of patients that survive after 5 years from diagnosis [2,3], hence the need to test new drugs for improving therapeutic strategies. Nowadays, despite biological therapies that have revolutionized the prognosis of several types of tumors, chemotherapy still represents the gold standard treatment for patients affected by PC [4,5]. In fact, targeted therapies and immunotherapy have been unable to provide a significant survival improvement to PC patients [6]. This ineffectiveness could be explained by the specific features of the tumor microenvironment (TME) in PC. The knowledge about the roles of all involved elements in the TME, their complex interactions, and the mechanisms that lead to treatment resistance and cancer immune escape mechanisms is a fundamental requisite to develop new immunological therapeutic strategies against pancreatic tumor cells [7]. In addition, it is important to identify predictive factors of the response to immunotherapy with the aim of selecting those patients that might benefit from these therapies. In this review, we focused on the immunological aspects of the pancreatic TME and their role in therapeutic strategies. Moreover, we also discussed the potential value of predictive and prognostic immune factors. Finally, we provided an up-to-date overview of immunotherapy for PC.

## 2. The Pancreatic Tumor Microenvironment

**Figure 1:**
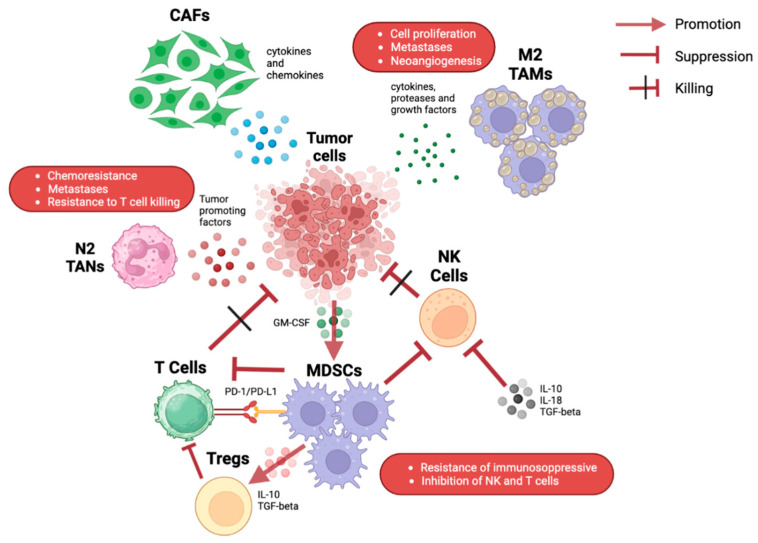
Illustration of the various possible interactions among immune cells in the pancreatic tumor microenvironment. CAFs: cancer-associated fibroblasts; GM-CSF: granulocyte-macrophage colony-stimulating factor; MDSC: myeloid-derived suppressor cells; NK: natural killer; PD-1: programmed death-1; PD-L1: programmed death-ligand 1; TAMs: tumor-associated macrophages; TANs: tumor-associated neutrophils.

PC has a rich and dense stroma, in which several types of cells are present such as fibroblasts, immune cells, stellate cells, and endothelial cells [8]. The complex signals between these cells and tumor cells are responsible for tumor proliferation and survival and/or the response or resistance to drugs. Various immune cells are present in the pancreatic TME in different proportions. These cells can interact with each other, leading to various effects (Figure 1) [9,10].

### 2.1. Tumor-Associated Macrophages

Myeloid cells correspond to the most represented cellular component in the stroma. Literature data correlate the high number of tumor-associated macrophages (TAMs) with poor prognosis in PC patients [11,12]. TAMs might exist in two different phenotypes with opposite functions: M1-like macrophages release pro-inflammatory cytokines with anti-tumor effects while M2-like macrophages have anti-inflammatory effects. M2-like macrophages produce several proteases, cytokines, and growth factors leading to cancer cell proliferation, neo-angiogenesis, and metastases [13,14,15,16].

### 2.2. Myeloid-Derived Suppressive Cells

Pancreatic tumor cells can recruit Myeloid-derived suppressive cells (MDSCs) in the pancreatic TME by means of the production of the granulocyte-macrophage colony-stimulating factor (GM-CSF) [17,18]. MDSCs exert an important anti-inflammatory action through the inhibition of both innate and adaptive immunity systems. In particular, MDSCs may employ a direct-contact mechanism for blocking natural killer cells (NK cells) and favoring the suppression of T-cell activation through the upregulation expression of programmed death-1 (PD-1) on its surface [19,20,21]. In addition, MDSCs can recruit immunosuppressive regulatory T cells, called Tregs, by producing transforming growth factor-beta (TGF-beta) and interleukin-10 (IL-10) [22].

### 2.3. Natural Killer Cells

NK cells play a pivotal role through direct killing action on cancer cells. This function is not related to antigen stimulation, but it is based on several types of cell receptors, including natural cytotoxicity receptors (NCRs), natural killer group 2 membrane D (NKG2D), 90 CD16, and DNAM-1 [23,24].

However, NK cells have a low and impaired activity in the pancreatic TME due to complex interactions with tumor cells and other immune cells. These NK cells produce a very low number of proteases such as perforin and granzyme B, and they are characterized by a lower surface expression of the chemokine receptor CXCR2 [25,26]. 

The main causes of this low-function state of NK cells correspond to the over-production of IL-18, IL-10, and TGF-beta, and to the downregulation of activating receptors [27,28].

### 2.4. Tumor-Associated Neutrophils

Neutrophils in the TME are called tumor-associated neutrophils (TANs). In the pancreatic TME, they may be present in two different states: N1 neutrophils that are polarized by TGF and N2 neutrophils by IFN [29]. The first ones have pro-inflammatory functions; indeed, they promote the chemotaxis and activation of CD8+ T cells [30]; the second ones have a pro-tumor action through the release of several types of proteases including neutrophil elastase (NE), metalloproteinase (MMPs), and other tumor-promoting factors such as reactive oxygen and nitrogen species [31,32]. Neutrophils can also produce a type of adipokine called lipocalin-2, which is involved in cancer cell activation and stromal remodeling [33].

Tregs can produce IL-17 that can indirectly induce the production of neutrophil extracellular traps (NETs) [34]. The formation of NETs may block CD8+ T cells and favor the occurrence of immune checkpoint inhibitor (ICI) resistance and liver metastasis.

### 2.5. T-Regs

T regs have numerous anti-inflammatory functions, mainly due to the suppression of T-112 cell function. Moreover, they are involved in the early phases of carcinogenesis [35]. In this regard, a lot of Th-17 cells and Treg are present in premalignant lesions, including pancreatic intraepithelial neoplasia (PanIN) and intraductal papillary mucinous neoplasm (IPMN) [36,37,38]. In addition, an elevated Th2/Th1 tumor-infiltrating lymphocyte ratio has been discovered in the pancreatic TME [38,39,40]. Various stimulating factors produced by different immune cells such as B cells, dendritic cells, TAMs, cancer-associated fibroblasts (CAFs), dendritic cells, and TAMs favor Th2 enrichment in the pancreatic TME [41,42,43]. Th2 lymphocytes exert their role through GATA3, which stimulates M2 macrophage activation and induces tumor cell proliferation. The pro-tumor function is also strictly correlated with the elevated activation of AKT, STAT3, and MAPK pathways [44].

### 2.6. CD8+ T Lymphocytes

Although CD8+ T cells lymphocytes usually exert a direct and cytotoxic activity on tumor cells, the TME may lead to CD8+ T exhaustion of this type of lymphocyte, with subsequent impaired cytotoxic actions and shorter cell survival [45]. T cell exhaustion corresponds to a progressive loss of effector activity (loss of TNF-α, IL-2, and IFN-γ production) and increased expression of inhibitory receptors such as CD160, the T cell immunoglobulin domain, lymphocyte-activation gene 3 (LAG-3), PD-1, and CTLA-4 [46]. The interactions between immune system and tumor cells in the TME led to a change in differentiation of CD8+T lymphocytes [47]. A lot of tumor cells express self-antigens, depleting many partial tumor-specific T lymphocytes during thymic maturation, while the other tumor-specific T cells have a low affinity for antigen recognition [47,48]. Moreover, the TME is characterized by the lack of innate stimulators, so antigen-presenting cells (APCs) are weakly activated, leading to a suboptimal activation of tumor-specific T lymphocytes [49]. In addition, on the one hand, immune cells kill tumor cells but, on the other hand, tumor cells recruit immunosuppressive cells, generating the immunosuppressive TME [50,51].

### 2.7. No-Immune Cells

Many types of non-immune cells such as CAFs and stellate cells can be found in the TME with very important roles [52,53,54]. In detail, three different CAF subpopulations have been identified. The first one is close to tumor cells and has myofibroblastic and anti-tumor effects. TGF stimulates this subpopulation, polarizes macrophages to M2 subtypes, suppresses T-cell activity, and is involved in the production of extracellular matrix, in the epithelial-to-mesenchymal transition, and in cell growth [55,56,57,58]. On this basis, Galunisertib, a TGF inhibitor, has been evaluated in combination with chemotherapy as the first-line treatment of PC [59]. The second subpopulation favors an immunosuppressive state of the TME following the IL-1-mediated activation [56]. The expression of IL-1 can be increased by gut commensal or intra-tumoral bacteria with the consequent activation of this type of CAF. This last subpopulation can produce several factors such as IL-33, IL-6, IL-8, and CXCL12, which in turn promote tumor angiogenesis and provide chemoresistance and resistance to the T-cell killing effect [60,61,62]. The third ones, called antigen-presenting CAFs, are the third subpopulations and their role has not been well defined yet, although it seems that they probably favor an immune-suppressive state [54]. Finally, the microbiota can significantly influence the immunological state of the pancreatic TME. In vivo studies on murine models reported that bacterial ablation led to a lower level of M2 macrophages, a larger number of CD8+ T-lymphocytes, and a higher expression level of PD-1 in the pancreatic TME. Therefore, it is possible to assume that a therapeutic strategy including ICIs and antibiotics might be effective in this cancer, although a possible limiting factor might be represented by safety. Further clinical studies will be helpful to better evaluate this combination strategy.

## 3. Prognostic and Predictive Immune Biomarkers

Although the results reported with immunotherapy in PC are unfavorable, there is a small portion of PC patients that seem to benefit very much from this type of treatment [6], hence the importance of identifying specific biomarkers to predict the response to ICIs. The most important predictive factor of immunotherapy efficacy in PC corresponds to the presence of a high tumor mutational burden (TMB) since it is associated with a high level of cancer neoantigens [63]. However, a cut-off to indicate a TMB as “high” has not been defined yet; indeed, it varies according to studies and histology [64,65]; most studies regarding PC identified a cut-off of 20 mutations/Mb. PC is typically characterized by a very low median TMB, approximately 1–4 mutations/Mb, while PC with high TMB corresponds only to 1.1% of cases [66,67,68]. Note that the highest level of TMB has been detected in two very rare histological types (<2%), mucinous-colloid and medullary [69]. Furthermore, high-TMB PC is often (about 60%) associated with a mismatch repair deficiency (dMMR) or high microsatellite instability (MSI-H) and/or mutations in ERBB2, POLE, BRCA2, and BRAF genes [69]. Clinical data show that PC patients with high-TMB and MSI-H/dMMR experienced an important objective response rate (ORR) when treated with anti-PD-1 anti-bodies [69]. These data suggest a potential role of TMB as a potential predictive factor of response to immunotherapy for PC patients.

The expression level of programmed death-ligand 1 (PD-L1) is a well-defined predictive factor of ICI efficacy for several types of cancers. However, the number of cells expressing PD-1 and PD-L1 is lower in PC than those cancers where ICIs showed an important activity [70]. PD-L1 expression is present in around 30–40% of PC and is related to poor prognosis and a low presence of tumor-infiltrating lymphocytes, specifically CD8+ cells [71,72]. In addition, its expression seems to be favored by MLL1, MYC, and RAS mutations [73,74,75]. The literature data describe four PC models according to PD-L1 expression on immune cells (ICs) and tumor cells (TCs) [71]:

- Adaptive-1 (ICs > 1%, TCs: 0);
- Adaptive-2 (ICs > 1%, TCs > 1% to <25%);
- Constitutive (ICs: 0, TCs ≥ 25%);
- Combined (ICs > 1%, TCs ≥ 25%).

Tumors with an adaptive-1 pattern had a T-cell-inflamed TME, a high level of PD1+ T cells and CD3+, CD4+, and CD8+ lymphocytes, and a reduced number of CD68+ cells, including the M2-polarized macrophages. This pattern is correlated with the longest survival. On the other hand, tumors with constitutive patterns are characterized by a low number of ICs, except for CD68+ macrophages, and are associated with poor clinical results. In terms of prognostic role, the immune infiltration of CD163+ M2-polarized macrophages in PC has a negative impact while an elevated infiltration of CD4+ and CD8+ cells is correlated with a longer disease-free survival [72]. Currently, the presence of MSI-H/dMMR is the only factor proved to predict the response to ICIs for PC, although it regards only 3% of PC patients [76,77,78]. As mentioned before, the coexistence of high-TMB and MSI-H/dMMR occurs in 60% of PC and also shares several features, including a high prevalence of mucinous/colloid and medullary histology and a characteristic genomic landscape, with less common KRAS and TP53 mutations and more frequent JAK mutations with respect to microsatellite stable PC [78]. Mutant KRAS has a well-known oncogenic role, but it seems to be involved also in the formation of an immunosuppressed TME. In detail, KRAS mutations led to the downregulation of HLA class I on the cell surface and the overexpression of PD-L1 and CD47 with the consequent prevention of innate and adaptative anti-cancer responses [79,80,81].

In addition, mutant KRAS (mKRAS) promotes a paracrine network favoring TME infiltration with stromal cells and suppressive ICs and leading to a desmoplastic reaction in the TME [80,82]. Moreover, mKRAS favors TME infiltration by MDSCs and T-cell exclusion through the increased tumor expression of CXCL1 and GM-CSF, promotes the downregulation of CCL4 expression, impeding DC recruitment, and has a pro-inflammatory role by activating the Sonic Hedgehog signaling pathway and stimulating the expression of IL-6, COX2, MMP7, and pSTAT3 [17,83,84,85,86]. Experimental data on mouse models described as the inactivation of this tumor oncogene determined the formation of pancreatic intraepithelial neoplasia (PanIN) [87,88]. Moreover, PanIN lesions were infiltrated by MDSCs, TAMs, and Tregs. On the other hand, the level of type I conventional dendritic cells (cDCs-1) decreased as PanINs progressively evolved to invasive cancers [89,90]. These data correlate with the low number of infiltrating DCs found in human PC [91,92,93]. Exome studies have discovered that activating KRAS mutations are present in more than 50% of human PC [93,94]. Therefore, an increased level of mutated KRAS genes might represent a predictive factor of PC progression and metastasis [95,96]. On these bases, a novel therapeutic strategy might be the employment of the KRAS inhibitor to sensitize PC to immunotherapy [82]. In this regard, mouse cancer models tested the pharmacologic inhibition of KRAS G12C that led to an increased level of MHC-I expression on cancer cells and promoted TME infiltration by cDCs-1 and T-cells [97]. These events favored tumor sensibilization to immune modulation. However, KRAS G12C mutations only rarely occur in PC, and no targeted therapies have been approved for the other mKRAS variants, although some strategies, such as engineered exosomes and mKRAS-specific T lymphocytes, have shown interesting results [98,99].

## 4. Immunotherapy: State of the Art

Despite several ICIs, including anti-CTLA4, anti-PD-1, and anti-PD-L1 antibodies, documenting high efficacy in various types of cancers, the same is not true for PC. Indeed, the results derived from the administration of immunotherapy in PC patients have been largely disappointing [6,100].

Different efforts have been performed to improve the immune infiltration of the pancreatic TME. For example, it is well known that the inhibition of CXCR4 might result in increased T-cell chemotaxis. In this regard, preclinical modes demonstrated enhanced T-cell expansion and cancer cell death through the employment of PD-1 plus CXCR4 inhibition [101].

Another strategy to enhance the anticancer activity of the TME could be represented by CD40 activation. Indeed, agonistic CD40 antibodies have been proven to enhance tumor cells killing mediated by T lymphocytes and to rescue ICI sensitivity when combined with chemotherapy [102,103,104,105]. A phase I trial evaluated sotigalimab—a CD40 agonistic monoclonal antibody—and sotigalimab plus chemotherapy in combination or not with nivolumab—a PD-1 inhibitor—as a first-line therapy for metastatic PC patients. The results reported an ORR of 58% without serious adverse events (AEs) [105].

Two phase II clinical trials tested single-agent ICIs or their combination with the aim to improve immunotherapy activity on advanced PC. However, single-agent ipilimumab and durvalumab, anti-CTLA-4 antibodies, as single-agents or in combination with tremelimumab, a PD-L1 inhibitor, did not show significant results [106,107].

At the same time, the combination of a single ICI or dual immune blockade with chemotherapy (gemcitabine plus nab-paclitaxel) proved to be poorly effective [108,109]. The addition of pembrolizumab to chemoradiation therapy in the neoadjuvant setting did not lead to an improvement compared to chemoradiation alone and an enhanced immune infiltration in the TME [110]. A phase II pilot study analyzed nivolumab plus paricalcitol and chemotherapy (gemcitabine, cisplatin, and nab-paclitaxel) in 10 advanced PC patients as a first-line treatment. Although a very low number of patients was enrolled in this study, an ORR of 80% and a disease control rate (DCR) of 100% were obtained, but no further investigations were performed [111].

Another interesting strategy to improve immunotherapy efficacy is represented by the combination of ICI with vaccines. In this regard, a study tested ipilimumab plus GVAX, a granulocyte-macrophage colony-stimulating factor (GM-CSF) cell-based vaccine. The results showed an increased T-cell repertoire in the TME and better overall survival (OS) with respect to ipilimumab alone, with a statistically significant difference [112]. Another trial evaluated GVAX plus nivolumab or with nivolumab and urelumab, an anti-CD137 antibody, as a neoadjuvant or adjuvant treatment for resectable PC patients. The experimental group treated with GVAX, nivolumab, and urelumab obtained a better OS, disease-free survival (DFS), and pathologic response, but without a statistically significant difference [113]. A phase III clinical study investigated an allogenic vaccine, algenpantucel-L, made up of Gal-expressing engineered PC cell lines, combined with chemotherapy and chemoradiotherapy in the adjuvant setting. However, no significant improvements were observed [114,115].

Interestingly, oncolytic viruses have also been studied in PC, as single agents or combined with conventional treatments. They are natural or genetically modified viruses tested as treatment of different tumors. They may directly cause tumor cell killing or indirectly modify the TME indirectly favoring tumor regression [116,117,118]. Preclinical and clinical data show few promising results through the employment of reovirus, vaccinia, adenovirus, and herpes simplex 1. The poor results are probably due to the elevated density of the pancreatic TME that determines a low penetrability to viruses [119]. A phase II clinical trial tested the combination of Pelareorep, an isolate of a reovirus strain derivate, with gemcitabine. The results documented high viral replication in PC cells and an acceptable profile of safety [120]. A phase Ib trial evaluated Pelareorep plus chemotherapy and immunotherapy in pretreated PC patients reporting favorable outcomes and good tolerance [121]. Bruton tyrosine kinase (BTK) is expressed by various ICs [122]. In vivo data on PC murine models showed that the inhibition of BTK led to the differentiation of CD8 T-cells and the shift from an M2-like to M1-like macrophage phenotype [43]. In addition, when the BTK inhibition was associated with gemcitabine, an enhanced tumor shrinkage was determined [43]. In this regard, a randomized phase II clinical trial analyzed the combination of Acalabrutinib—a BTK inhibitor—combined or not with pembrolizumab, in pretreated PC patients. The combination group experienced a DCR of 29% with respect to 14.4% of the acalabrutinib-alone group [123].

Based on the favorable clinical results for those PC patients with an elevated T-lymphocytes infiltration, it is easy to assume that tumor infiltrating lymphocyte (TIL) therapy may represent a potential anti-cancer activity although most PC patients do not have pre-existing T-cell immunity, limiting this type of strategy [118,119]. The continuous research about this topic has rapidly led to very important results for patients suffering from hematological tumors through the employment of T-cell receptor T-cell (TCR-T) therapy and chimeric antigen receptor T-cell (CAR-T) therapy [120]. Unfortunately, these results were not obtained for solid tumors. However, different strategies have been tested to improve TIL therapy, for example, by means of the combination with immune modulating agents or the expression of transgenes to improve T-cell infiltration and activity [121]. The most important limitation to TCR-T and CAR-T treatments in PC corresponds to the antigen selection since they can have variable or heterogeneous expression on cancers cells, determining an elevated risk of toxicity. CEA, HER2, and mesothelin antigens have been mainly studied for CAR-T therapy in PC [122]. However, the employment of T lymphocytes directed against CEA and HER2 antigens determined serious adverse events [123,124,125]. On the other hand, mouse models [126] and early-phase clinical trials showed important activity and good safety by CAR-T engineered to target mesothelin in advanced PC patients pretreated with chemotherapy [127,128]. mKRAS has a high frequency in PC, so a neoantigen-targeted TCR therapy might represent a favorable strategy [93,129]. Currently, clinical studies are investigating this strategy [93].

Immunosuppressive ICs in the TME such as TAMs and MDSCs may represent a target of immune-based treatments with the aim of eliminating inhibitory elements that hamper T-cell responses. In this regard, CSF-1R inhibition favors antigen presentation and anti-cancer T-cell activity by TAMs that are suppressed by immune checkpoints [130]. Moreover, the combination of CSF-1R inhibition and ICI greatly promotes anti-tumor efficacy [130]. Early-phase clinical trials showed interesting results from the CSF-1R and CCR2 inhibition in advanced PC [76,131]. However, the combination of cabiralizumab, a CSF-1R blocking monoclonal antibody, with chemotherapy and nivolumab in metastatic PC patients did not improve PFS with respect to chemotherapy alone in a phase II study.

Another strategy is to target stromal elements, such as hyaluronan, the vitamin D receptor (VDR), focal adhesion kinase (FAK), and fibroblast activation protein (FAP), that promote the desmoplastic reaction and the consequent chemo-resistance of PC [132]. PEGPH20 is a hyaluronidase that has been evaluated in combination with chemotherapy compared to chemotherapy alone for untreated metastatic PC patients with high hyaluronan levels in a phase III trial [133]. However, the results did not demonstrate an improvement of OS. PEGPH20 has also been examined in association with mFOLFIRINOX in a phase II trial [134]. However, the study was prematurely closed because of poor results and elevated toxicity. These unsatisfactory outcomes suggest that targeting stromal elements alone is insufficient to overcome PC immune resistance. Currently, treatment strategies that combine ICIs plus targeting desmoplasia are ongoing. Early-phase clinical studies are currently under investigation with the aim to test FAK inhibitors plus αPD1 antibodies [135]. Moreover, FAP-directed CAR-T therapy showed an anti-cancer response in tumor mouse models [136,137].

As mentioned before, MSI-H is present in 1–2% of PC, and high TMB is very rare in this tumor [78,138,139]. The KEYNOTE-158 trial investigated pembrolizumab in patients affected by advanced non-colorectal cancers with MSI-H/MMR-d including 22 PC patients. This group of patients experienced an ORR of 18.2%, a median duration of response of 13.4 months, and a PFS and OS of 2.1 and 4.0 months, respectively. However, these results were considerably lower than patients who suffered from other tumors [64]. Table 1 summarizes all ongoing clinical studies evaluating immunotherapy in PC patients.

## 5. Conclusions

To date, no clinical trial testing immunotherapy or targeted therapy for PC patients has led to practice-changing results. This is probably due to the peculiar pancreatic TME and its genomic landscape. Further studies are needed to better understand TME features that make PC resistant to immunotherapy with the aim to design modification mechanisms so that PC becomes more immunosensitive. A better selection of patients that might benefit more from immunotherapy is also required. An useful approach could derive from the identification of biomarkers able to predict clinical outcomes following the administration of immunotherapy.

## Figures and Tables

**Table 1:** Ongoing clinical studies evaluating immunotherapy in PC patients.

Immune Checkpoint Inhibitors	NCT	Phase	Setting	Comparison	Primary Endpoints
	NCT04548752	I/II	Maintenance in BRCA- mutated patients	Pembrolizumab + Olaparib vs. Olaparib	PFS
	NCT05093231	II	First-line	Pembrolizumab + Olaparib	ORR
	NCT04753879	II	First-line	Gemcitabine +Nab-paclitaxel + Cisplatin + Capecitabine + Irinotecan + Pembrolizumab + Olaparib as maintenance therapy	PFS
	NCT04887805	II	Maintenance therapy following first/second-line chemotherapy	Pembrolizumab + Lenvatininb	PFS
	NCT02648282	II	First-line	Pembrolizumab + Cyclophosphamide +GVAX + SBRT	DMFS
	NCT02305186	I/II	Neoadjuvant	chemoradiotherapy (with Capecitabine) vs. Pembrolizumab + chemoradiotherapy (with Capecitabine)	TILs per HPF Safety
Anti-PD-1 antibody	NCT02907099 [84]	IIb	First-line	Pembrolizumab + CXCR4 antagonist BL-8040	ORR
	NCT03727880	II	Neoadjuvant/Adjuvant	Pembrolizumab + Defactinib vs. Pembrolizumab	pCR
	NCT03977272	III	First-line	Anti-PD-1 antibody 200 mg + mFOLFIRINOX vs. mFOLFIRINOX	OS
	NCT03983057	III	First-line	Anti-PD-1 antibody 3 mg/Kg + mFOLFIRINOX vs. mFOLFIRINOX	PFS
	NCT03989310	I/II	First-line	PD-1 inhibitor + Manganese chloride + Gemcitabine + Nab-paclitaxel vs. PD-1 inhibitor + Nab-paclitaxel + Gemcitabine	DCR Safety
	NCT03161379 [96]	II	Neoadjuvant	Nivolumab + Cyclophosphamide +GVAX + SBRT	CD8 count in TME
	NCT04377048	II	First-line	Nivolumab + Gemcitabine + Tegafur-Gimeracil-Oteracil	ORR
	NCT03563248	II	Neoadjuvant	FOLFIRINOX → SBRT → Surgery vs. FOLFIRINOX + Losartan → SBRT + Losartan → Surgery vs. FOLFIRINOX + Losartan → SBRT + Nivolumab + Losartan → Surgery vs. FOLFIRINOX → SBRT + Nivolumab → Surgery	R0
	NCT03767582	I/II	Locally advanced	Nivolumab + CCR2/CCR5 dual anta-gonist + SBRT vs. Nivolumab+ CCR2/CCR5 dual anta-gonist + GVAX + SBRT	ORR Safety
	NCT04543071	II	First-line	Cemiplimab, Motixafortide, Gemcitabine, Nab-Paclitaxel	ORR
	NCT04177810	II	First-line	Cemiplimab + Plerixafor	ORR
	NCT04493060	II	First-line	Dostarlimab + Niraparib	DCR
Anti-PD-L1 antibody	NCT04940286	II	Neoadjuvant	Oleclumab + Durvalumab + Gemcitabina + Nab-Paclitaxel	mPR
Anti-CTLA4 antibody	NCT04827953	I/II	First-line	Zalifrelimab + Gemcitabine + Nab-Paclitaxe + NLM-001	ORR
	NCT04156087 [90]	II	Locally advanced	Durvalumab + MIS-MWA + Tremelimumab	PFS
	NCT05116917	II	First-line	SBRT + Nivolumab + Influenza vaccine + Ipilimumab	ORR
	NCT03193190	Ib/II	First-line	Atezolizumab, Gemcitabine, Nab-Paclitaxel, Fluorouracil, Oxali-platin, PEGPH20, Cobimetinib, BL-8040, AB928, RO6874281, Bevacizu-mab, Selicrelumab, Tocilizumab, and Tiragolumab	ORR Safety
Combination of ICIs	NCT04361162	II	First-line	Nivolumab + Ipilimumab + Radiation	ORR
	NCT03336216	II	Second-line or later	5-FU/Leucovorin/Irinotecan Liposome or Nab-Paclitaxel/Gemcita-bine vs. Nivolumab + Cabiralizumab vs. Gemcitabine + Nab-Paclitaxel + Cabiralizumab + Nivolumab vs. Cabi-ralizumab + Nivolumab + FOLFOX	PFS
	NCT04247165	I/II	Locally advanced	Nivolumab + Gemcitabine + SBRT+ Nab-Paclitaxel + Ipilimumab	Safety
	NCT05014776	II	Second-line or later	Pembrolizumab + Ipilimumab + Tadalafil + CRS-207	irORR
	NCT03190265	II	Second-line or later	Nivolumab +GVAX + CRS-207 + Ipilimumab + Cyclophosphamide vs. Nivolumab + CRS-207 + Ipilimumab	ORR

PD-1: programmed death-1; CTLA-4: Cytotoxic T-Lymphocyte Antigen 4; PD-L1: programmed death-ligand 1; DCR: disease control rate; DMFS: distant metastasis-free survival; OS: overall survival; PFS: progression-free survival; irORR: Objective response rate using immune Response Evaluation Criteria for Solid Tumors; HPF: high-powered field; pCR: pathologic complete response; CCR2: Chemokine (C-C motif) receptors 2; CXCR4: C-X-C Motif Chemokine Receptor 4; CCR5: Chemokine (C-C motif) receptors 5; TILs: tumor-infiltrating Lymphocytes; GVAX: granulocyte-macrophage colony-stimulating factor (GM-CSF) gene-transfected tumor cell vaccine; SBRT: Stereotactic Body Radiation Therapy; MIS-MWA: Minimally Invasive Surgical Microwave Ablation.

## Data Availability

Not applicable.
